# Risk factors for necrotizing enterocolitis in neonates: A meta-analysis

**DOI:** 10.3389/fped.2022.1079894

**Published:** 2023-01-06

**Authors:** Yan Su, Rui-Hong Xu, Li-Yan Guo, Xin-Qing Chen, Wen-Xiao Han, Jin-Jin Ma, Jiao-Jiao Liang, Ling Hao, Chang-Jun Ren

**Affiliations:** Department of Pediatrics, The First Hospital of Hebei Medical University, Shijiazhuang, China

**Keywords:** necrotizing enterocolitis, risk factor, meta analysis, neonate, newborn

## Abstract

**Objective:**

The objective is to identify the risk factors for necrotizing enterocolitis (NEC) in neonates by a meta-analysis, and to provide a reference for the prevention of NEC.

**Methods:**

The databases, including Chinese Biomedical Literature Datebase, China National Knowledge Infrastructure, Wanfang database, and Weipu Periodical database, PubMed, Web of Science, Embase, Cochrane Library, were searched for studies on the risk factors for NEC in neonates. The meta-analysis was carried out with the aid of Stata software.

**Results:**

A total of 52 studies were included, with 48 case-control studies and 4 cohort studies. There were 166,580 neonates in total, with 33,522 neonates in the case group and 133,058 neonates in the control group. The meta-analysis showed that gestational diabetes (OR = 3.62, 95% CI:1.77–7.41), premature rupture of membranes (OR = 3.81, 95% CI:1.16–12.52), low birth weight (OR = 3.00, 95% CI:2.26–3.97), small for gestational age (OR = 1.85, 95% CI:1.15–2.97), septicemia (OR = 4.34, 95% CI:3.06–6.15), blood transfusion (OR = 3.08, 95% CI:2.16–4.38), congenital heart disease (OR = 2.73, 95% CI:1.10–6.78), respiratory distress syndrome (OR = 2.12, 95% CI:1.24–3.63), premature birth (OR = 5.63, 95% CI:2.91–10.92), pneumonia (OR = 4.07, 95% CI:2.84–5.82) were risk factors for NEC in neonates. Breastfeeding (OR = 0.37, 95% CI:0.23–0.59), take probiotics (OR = 0.30, 95% CI:0.22–0.40), prenatal use of glucocorticoids (OR = 0.39, 95% CI:0.30–0.50), Hyperbilirubinemia (OR = 0.28, 95% CI:0.09–0.86) were protective factors for NEC in neonates.

**Conclusions:**

Gestational diabetes, premature rupture of membranes, low birth weight, small for gestational age, septicemia, blood transfusion, congenital heart disease, respiratory distress syndrome, premature birth, and pneumonia may increase the risk of NEC in neonates. Breastfeeding, taking probiotics, prenatal use of glucocorticoids, and Hyperbilirubinemia may reduce the risk of NEC in neonates.

## Introduction

Necrotizing enterocolitis (NEC) in neonates is a severe muti-factorial disease characterized by intestinal necrosis in the ileum, jejunum, and colon. It is one of the leading causes of morbidity and mortality in preterm infants (1), with which clinical manifestations involve abdominal distension, vomiting, bloody stool, septic shock, and DIC in severe cases. Therefore, early diagnosis and treatment to avoid its devastating consequences are essential. However, due to the poor insight into its pathogensis, reliable tools and effective strategies are short to prevent and treat NEC in neonates (2). Indeed, there are many pathogenic factors of NEC in neonates, some of which still need to be clearly defined. Since the identification and intervention of neonates at risk for NEC can reduce the incidence and improve the prognosis (3), this study comprehensively searched domestic and foreign literature on risk factors for NEC in neonates by a meta-analysis, which aims to provide a reference for the prevention of NEC in neonates.

## Materials and methods

### Document retrieval

China National Knowledge Infrastructure (CNKI), Wanfang Database, VIP Chinese Journal Database, Chinese Biomedical Literature Database, PubMed, Web of Science, Embase and Cochrane Library were searched to systematically collect published studies on risk factors of NEC in neonates. The search strategy of combining keywords and subject terms was adopted. The Chinese search terms were “newborn”, “necrotizing enterocolitis”, “risk factors”, “case-control study”, “cohort study”, etc. English search words “Enterocolitis, Necrotizing”, “Infant, Newborn”, “relative risk”, “cohort”, etc., supplemented by manual search and literature tracing.

### Inclusion and exclusion criteria

Inclusion criteria: 1. The diagnosis of NEC is clear; 2. The study type was a case-control study or cohort study; 3. The subjects were neonates (<28 days); 4. The original data is available. The OR (odds ratio) value and 95% confidence interval (CI) are provided, or the OR value and 95% CI can be calculated from the data.

Exclusion criteria: 1. Conference summary, comments and review articles; 2. Unable to extract effective outcome indicators from the literature; 3. The experimental design is not rigorous (non case-control or cohort studies, as well as grouping non case-control/exposure group and control group/unexposed group); 4. Unable to get a full text; 5. The sample size is too small.

### Data extraction and quality assessment

Two researchers strictly followed the inclusion and exclusion criteria to independently conduct literature screening, quality evaluation, data extraction, and discuss possible differences to reach an agreement. The final results were confirmed by more senior researchers. The Newcastle-Ottawa Scale (NOS) (4) was used for the quantitative assessment of case-control studies and cohort studies, including research object evaluation (4 points), inter group comparability evaluation (2 points), and result evaluation (3 points). The NOS score ≥ 6 is a high-quality study.

### Method of statistics

The Q test and I^2^ statistic were used to evaluate the heterogeneity, and the test level was set as 0.1. If the heterogeneity test results were *P* > 0.1 and *I*^2^ < 50%, the pooled effect size OR and 95% CI were calculated using the fixed effects model. Otherwise, the random effect model is used to calculate; Sensitivity analysis uses different models to analyze the same data. Egger's or Begg's test was used to evaluate publication bias, and Stata12.0 was used for statistical analysis.

## Result

### Literature screening results

**Figure 1:**
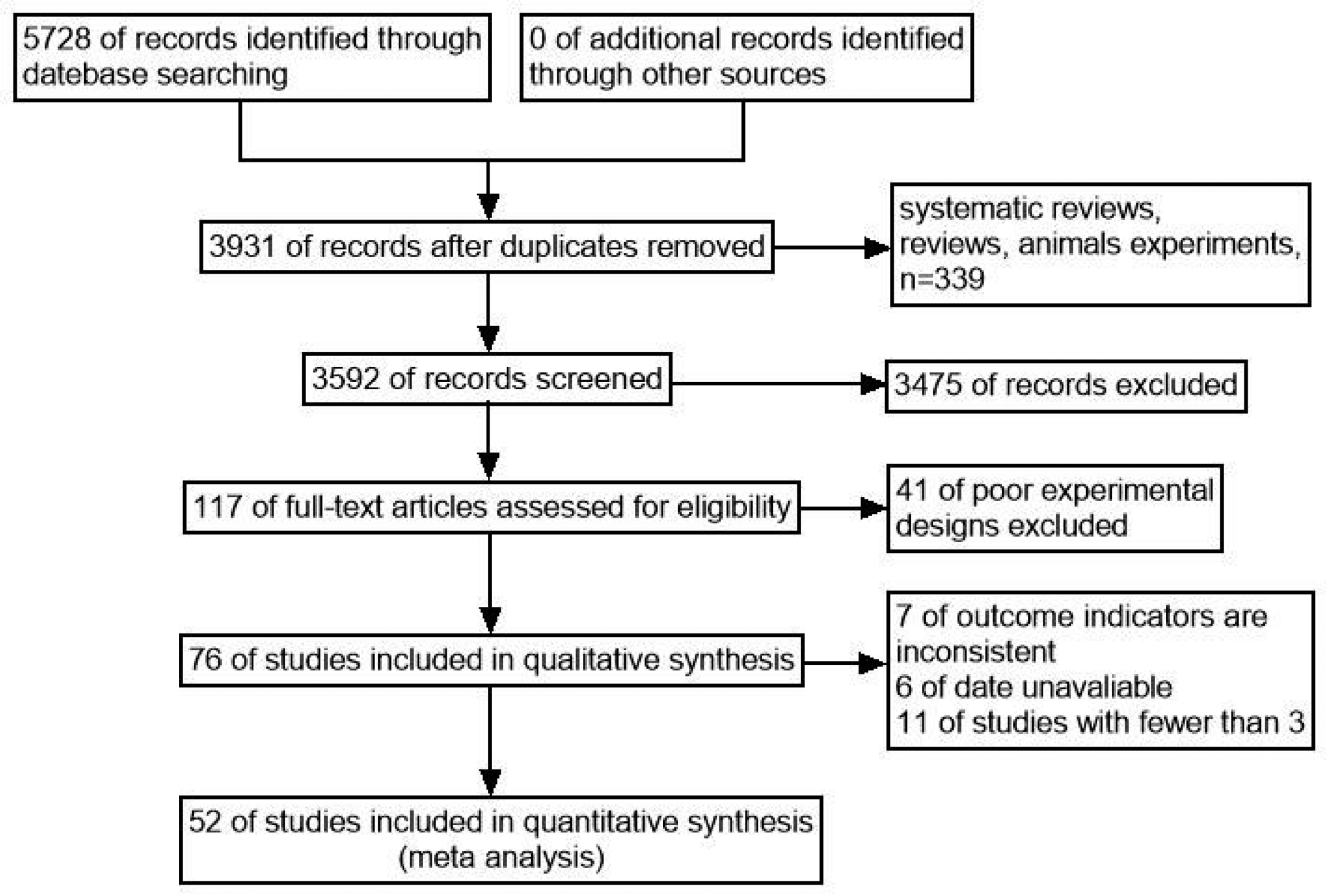
Flow chart of document screening.

A total of 5,728 relevant articles were obtained through preliminary screening, and 3,931 articles remained after being re-selected by Endnote software. Ulteriorly, 3,475 articles were excluded after reading the title and abstract, and 117 articles remained for further evaluation. Finally, after reading the full text, 52 articles were included according to the inclusion and exclusion criteria, as shown in Figure 1. There were 166,580 subjects, including 33,522 cases in the case group and 133,058 cases in the control group. 48 case-control studies and 4 cohort studies are shown in Table 1. The NOS score of 52 included studies was no less than 6 points.

**Table 1:** Characteristics of included studies.

Literature	Research type	Case group (exposure group)	Control group (non exposed group)	NOS score
Ahle M, et al. 2018 (5)	Case control	720	3656	6
Cetinkaya M, et al. 2017 (6)	Case control	26	119	6
Chen S, et al. 2020 (7)	Cohort study	30	150	8
Son M 2016 (8)	Cohort study	731	1281	8
Tan X, et al. 2022 (9)	Case control	68	124	8
Teišerskas J 2019 (10)	Case control	54	54	7
Valentine GC, et al. 2019 (11)	Cohort study	338	238	8
Yang CC, et al. 2018 (12)	Cohort study	29,013	116,052	9
Zhang LP, et al. 2019 (13)	Case control	33	33	8
Zeng DF 2017 (14)	Case control	59	80	6
Ceng SY 2021 (15)	Case control	30	467	7
Chen W 2021 (16)	Case control	25	25	6
Cheng SP 2016 (17)	Case control	66	132	7
Cui GH 2019 (18)	Case control	70	140	6
Fan WT 2019 (19)	Case control	69	100	6
Fan YZ 2015 (20)	Case control	63	70	7
Hou AN 2017 (21)	Case control	76	80	6
Huang YQ 2017 (22)	Case control	26	534	6
Jiang CC 2020 (23)	Case control	70	84	6
Ke H 2017 (24)	Case control	32	96	6
Li HY 2014 (25)	Case control	98	80	6
Li LQ 2006 (26)	Case control	20	80	7
Li MQ 2019 (27)	Case control	28	28	6
Li XH 2019 (28)	Case control	25	175	7
Liu YX 2021 (29)	Case control	46	1127	6
Liu YC 2019 (30)	Case control	95	2196	7
Lu XY 2013 (31)	Case control	54	57	7
Lu Y 2022 (32)	Case control	52	52	7
Lu M 2015 (33)	Case control	59	95	7
Ma J 2019 (34)	Case control	41	383	6
Ma XJ 2021 (35)	Case control	54	106	6
Ma ZX 2017 (36)	Case control	36	182	6
Shang Y 2014 (37)	Case control	37	62	7
Shi Y 2019 (38)	Case control	32	150	7
Sun HX 2017 (39)	Case control	37	60	7
Tao Y 2012 (40)	Case control	63	126	6
Wang B 2019 (41)	Case control	50	72	6
Wang J 2021 (42)	Case control	95	95	6
Wang PP 2020 (43)	Case control	30	34	7
Wang WH 2013 (44)	Case control	51	120	7
Wang XQ 2017 (45)	Case control	58	36	6
Wang Y 2017 (46)	Case control	85	2678	6
Wang ZQ 2020 (47)	Case control	42	38	7
Xi E 2017 (48)	Case control	60	60	6
Xu LY 2018 (49)	Case control	247	191	7
Yu M 2018 (50)	Case control	146	146	7
Zhang L 2017 (51)	Case control	61	376	6
Zhao XH 2017 (52)	Case control	46	78	6
Zhou XM 2017 (53)	Case control	67	73	7
Zhu JL 2020 (54)	Case control	38	462	6
Zhuang XY 2007 (55)	Case control	20	80	6
Zou YM 2021(56)	Case control	50	45	6

### Results of meta-analysis

According to the risk factors involved in the included literature, gestational diabetes, premature rupture of membranes, cesarean section, low birth weight, small for gestational age, sepsis, blood transfusion, congenital heart disease, respiratory distress syndrome, mechanical ventilation, breast feeding, probiotics, preterm delivery, pneumonia, prenatal use of glucocorticoids, hyperbilirubinemia, ect., were selected for analysis. Heterogeneity test results showed that there was heterogeneity among the studies of diabetes in pregnancy, premature rupture of membranes, cesarean section, small for gestational age, sepsis, blood transfusion, congenital heart disease, respiratory distress syndrome, mechanical ventilation, breast-feeding, probiotics, preterm delivery, pneumonia and hyperbilirubinemia, and the random effect model was used to combine the effect amount. In contrast, there is no heterogeneity in other related factors, and the fixed effect model is used to combine the effects. The meta-analysis results demonstrates that: Cesarean section and mechanical ventilation were not statistically significant with NEC in neonates. Gestational diabetes mellitus, premature rupture of membranes, low birth weight, small for gestational age, sepsis, blood transfusion, congenital heart disease, respiratory distress syndrome, premature birth and pneumonia were risk factors for NEC in neonates. Breastfeeding, probiotics, prenatal glucocorticoid use, and hyperbilirubinemia were protective factors for NEC in neonates, as shown in Table 2.

**Table 2:** Heterogeneity test and meta-analysis results of risk factors.

Factor	Number of studies	Heterogeneity test		Effect model	Pooled OR and 95% CI	Pooled *p* value
		*p* value	*I*²			
Gestational diabetes	6	*p* < 0.05	80.6%	Random effect	3.62 (1.77–7.41)	*p* < 0.05
Premature rupture of membranes	5	*p* < 0.05	93.1%	Random effect	3.81 (1.16–12.52)	*p* < 0.05
Cesarean section	4	*p* < 0.05	80.7&	Random effect	1.31 (0.86–2.00)	0.207
Low birth weight	8	0.238	23.1%	Fixed effect	3.00 (2.26–3.97)	*p* < 0.05
Small for gestational age	5	*p* < 0.05	66.4%	Random effect	1.85 (1.15–2.97)	*p* < 0.05
Septicemia	32	*p* < 0.05	88.9%	Random effect	4.34 (3.06–6.15)	*p* < 0.05
Blood transfusion	10	*p* < 0.05	54.0%	Random effect	3.08 (2.16–4.38)	*p* < 0.05
Congenital heart disease	4	*p* < 0.05	92.8%	Random effect	2.73 (1.10–6.78)	*p* < 0.05
Respiratory distress syndrome	5	*p* < 0.05	79.8%	Random effect	2.12 (1.24–3.63)	*p* < 0.05
Mechanical ventilation	4	*p* < 0.05	84.4%	Random effect	2.55 (0.98–6.61)	0.054
Breastfeeding	21	*p* < 0.05	94.2%	Random effect	0.37 (0.23–0.59)	*p* < 0.05
Take probiotics	28	*p* < 0.05	84.7%	Random effect	0.30 (0.22–0.40)	*p* < 0.05
Premature birth	7	*p* < 0.05	91.4%	Random effect	5.63 (2.91–10.92)	*p* < 0.05
Pneumonia	14	*p* < 0.05	54.7%	Random effect	4.07 (2.84–5.82)	*p* < 0.05
Prenatal use of glucocorticoids	7	0.345	11.1%	Fixed effect	0.39 (0.30–0.50)	*p* < 0.05
Hyperbilirubinemia	6	*p* < 0.05	92.3%	Random effect	0.28 (0.09–0.86)	*p* < 0.05

### Sensitivity analysis and bias test

For the screened risk factors, the fixed effect and random effect models were used to recalculate the combined effect size. The calculation results of these two models were basically consistent, indicating that the results of this study were basically reliable. However, the results of premature rupture of membranes are not robust enough and should be treated with caution. The results of Egger's or Begg's test suggest that the results of sepsis, probiotics, and pneumonia are biased (*P* < 0.05), as shown in Table 3. The pruning method evaluated the publication bias of the results of sepsis, probiotics and pneumonia. It was found that the results of sepsis [OR and 95% CI: 2.13 (1.47–3.10)], probiotics [OR and 95% CI: 0.30 (0.22–0.41)], and pneumonia [OR and 95% CI: 2.67 (1.82–3.92)] were basically consistent before and after pruning, suggesting that the meta-analysis conclusion of risk factors was stable and reliable.

**Table 3:** Sensitivity analysis and bias test.

Factor	Sensitivity analysis		Egger's Test/Begg's Test
	Fixed effect model (pooled OR and 95% CI)	Random effect model (pooled OR and 95% CI)	*p* value
Gestational diabetes	4.62 (3.46–6.16)	3.62 (1.77–7.41)	0.254
Premature rupture of membranes	0.99 (0.80–1.22)	3.81 (1.16–12.52)	0.806
Low birth weight	3.00 (2.26–3.97)	3.02 (2.18–4.19)	0.076
Small for gestational age	1.34 (1.08–1.67)	1.85 (1.15–2.97)	0.260
Septicemia^a^	3.03 (2.73–3.36)	4.34 (3.06–6.15)	0.028
Blood transfusion	2.55 (2.06–3.16)	3.08 (2.16–4.38)	0.210
Congenital heart disease	1.09 (1.03–1.15)	2.73 (1.10–6.78)	0.089
Respiratory distress syndrome	1.38 (1.15–1.66)	2.12 (1.24–3.63)	0.221
Breastfeeding	0.52 (0.48–0.57)	0.37 (0.23–0.59)	0.393
Take probiotics^b^	0.65 (0.60–0.71)	0.30 (0.22–0.40)	0.017
Premature birth	6.13 (5.19–7.22)	5.63 (2.91–10.92)	0.697
Pneumonia^c^	3.37 (2.68–4.24)	4.07 (2.84–5.82)	0.004
Prenatal use of glucocorticoids	0.39 (0.30–0.50)	0.38 (0.28∼0.51)	0.072
Hyperbilirubinemia	0.70 (0.54–0.92)	0.28 (0.09–0.86)	0.181

^a^ indicates that the OR and 95% CI after correction are 2.13 (1.47–3.10).

^b^ indicates that the OR and 95% CI after correction are 0.30 (0.22–0.41).

^c^ shows that the corrected OR and 95% CI are 2.67 (1.82–3.92).

## Discussion

This study conducted a meta-analysis of the domestic and foreign studies on the risk factors of NEC in neonates, and conducted a quantitative combined analysis and comprehensive evaluation of the results of multiple studies with the same research factors, to make the research conclusions more comprehensive and reliable.

This meta-analysis shows that gestational diabetes is a risk factor for NEC in neonates. As the nutrition needed for the development of the fetus in the abdomen comes from the mother, the blood sugar of the mother after diabetes during pregnancy is higher than the normal level, and high blood sugar will inhibit the blood circulation of the fetus' intestinal tract, causing ischemic necrosis of the intestinal mucosa. After birth, pathogenic microorganisms easily invade the gastrointestinal tract and colonize the damaged intestinal mucosa, causing inflammation and morbidity (57).

This meta-analysis shows that premature rupture of membranes may increase the risk of NEC. Research shows that PROM, as one of the main causes of premature delivery, can increase the incidence of NEC, ROP, BPD and other complications. Timely treatment of PROM can reduce the occurrence of NEC in neonates (58).

This meta-analysis showed that low birth weight was a risk factor for NEC. The reasons may be as follows: (1) Due to the immature intestinal function and slow intestinal peristalsis of low birth weight infants, food residues are easy to be detained and fermented, providing a good environment for bacterial growth, leading to a large number of bacterial proliferation; (2) The intestinal microbiota of low birth weight infants is very immature, and direct contact with pathogenic microorganisms will cause inflammation related to mucosal damage, leading to NEC (59).

This meta-analysis showed that small for gestational age (SGA) was a risk factor for NEC. SGA infants have a high probability of NEC, neonatal asphyxia, brain injury and respiratory distress syndrome (60). One study found that the risk of NEC in SGA infants was twice that of appropriate for gestational age infants (61).

This meta-analysis showed that septicemia, congenital heart disease, respiratory distress syndrome, and pneumonia were risk factors for NEC. In severe infection, the body is in a state of inflammation activation, producing a variety of inflammatory transmitters. These substances directly or indirectly cause damage to the intestinal mucosa, and then participate in the occurrence and development of NEC (62, 63). Meanwhile, the combined effects of pathogenic bacteria, intestinal flora imbalance, intestinal wall barrier dysfunction, toxic intestinal paralysis, etc., during sepsis can also lead to NEC (64). In addition, in the case of sepsis and other severe infections, in addition to the direct destruction of intestinal epithelial cells by bacteria, endotoxin and other products produced by bacteria can also cause intestinal necrosis (62). In the previous report, the proportion of NEC in children with CHD was 6.8%∼13% (65), significantly higher than that in normal premature infants and neonates. Baxi et al. (66) found that children with CHD were prone to abnormal blood distribution, decreased mesenteric blood supply, and a large number of free radicals, which mediated reperfusion injury. It has been previously reported that asphyxia at birth is closely related to the occurrence of necrotizing enterocolitis. The severity of necrotizing enterocolitis increases with that of respiratory distress (67). When respiratory distress occurs or pneumonia occurs, the body is in an anoxic state. At this time, in order to ensure the oxygen supply of the vital organs of the child, the whole body's blood flow is redistributed, mainly because the intestinal vessels contract and the blood flow is reduced, leading to intestinal hypoperfusion, resulting in intestinal mucosa hypoxia and damage, leading to necrotizing enterocolitis (68, 69).

This meta-analysis showed that blood transfusion was a risk factor for NEC. The possible pathogenesis of NEC is as follows: the inflammatory mediators such as TNF-α, IL-6, and PAF produced during the processing of whole blood and the storage of red blood cells, and the residual white blood cells, free hemoglobin, red cell membrane fragments, etc. promote the occurrence of NEC. The pathological changes of red blood cells occurred during storage, including decreased erythrocyte deformability, increased oxygen affinity ability and decreased nitric oxide resulting in the loss of vasodilator activity, etc., resulting in the failure to improve intestinal microcirculation perfusion flow after blood transfusion; NEC may be caused by anemia (70).

This meta-analysis showed that preterm birth was a risk factor for NEC. Due to the unsound development of the enteric nervous system and poor regularity of small intestinal peristalsis, premature infants are prone to excessive bacterial growth and gas after food fermentation, and are prone to NEC (71).

This meta-analysis showed that breastfeeding, probiotics, prenatal glucocorticoid use, and hyperbilirubinemia were protective factors for NEC. Breast milk is known as the most natural and safe natural food for infants and young children, containing nutrients and antibodies necessary for the development of organized organs, especially beneficial antibodies, which can help maintain the immune function of newborns, inhibit the inflammatory reaction, and speed up the repair of the damaged intestinal mucosa (72). Compared with formula, breast milk has a lower osmotic pressure, which can minimize the osmotic load of food and reduce the impact on intestinal function, thereby reducing the incidence of NEC (73). The supplementation of probiotics may improve gastrointestinal tolerance (74). The raw materials of probiotics come from microorganisms that are beneficial to the body. In the intestinal tract of newborns, probiotics can play a role in improving the microecological balance and promoting intestinal peristalsis, which is of great significance in preventing the occurrence and development of neonatal necrotizing enterocolitis (75). Prenatal hormones can promote the generation of alveolar surfactants, contribute to the development of alveoli, and reduce the incidence of neonatal respiratory distress syndrome and mortality of preterm infants (76). Bilirubin is considered to have antioxidant activity, can scavenge free radicals in the body, and is one of the plasma free radical scavenger to defend against the damage of various oxides (77, 78).

## Limitations

This study has some limitations: First, sensitivity analysis found that the results of premature rupture of membranes were not robust enough. Therefore, the relationship between premature rupture of membranes and NEC in neonates needs further study. Secondly, there exist differences in sample size, case selection, and definition of exposure factors among the studies, which may lead to heterogeneity among the studies and have a certain impact on the results. Finally, only Chinese and English literature ware included in the included study, and literature published in other languages could not be analyzed, which may result in language bias.

## Conclusion

We conducted a meta-analysis to evaluate the risk factors of NEC. This meta-analysis showed that gestational diabetes mellitus, premature rupture of membranes, low birth weight, small for gestational age, sepsis, blood transfusion, congenital heart disease, respiratory distress syndrome, premature birth, and pneumonia maght increase the risk of NEC in neonates. Therefore, perinatal health care should be strengthened to reduce the incidence of neonatal complications, so as to prevent the occurrence of NEC in neonates.

## Data Availability

The original contributions presented in the study are included in the article/**Supplementary material**, further inquiries can be directed to the corresponding author/s.
