# Supplementary material for: Risk factors for necrotizing enterocolitis in neonates: A meta-analysis
Source: Front Pediatr. 2023 Jan 6;10:1079894. doi: 10.3389/fped.2022.1079894 (PMC9853297; doi:10.3389/fped.2022.1079894)
Supplement: Supplementary file 1 [file Table1.pdf]

Authors	Year of publication	Number of cases		main outcome	secondary outcomes
		Case group/ exposure group	Control group/ non exposure		
Ahle M, et al.	2018	720	3656	Independent factors related to NEC occurrence	-
Cetinkaya M, et al.	2017	26	119	Independent factors related to NEC occurrence	Occurrence of complications
Chen S, et al.	2020	30	150	Independent factors related to NEC occurrence	-
Son M	2016	731	1281	Independent factors related to NEC occurrence	-
Tan X, et al.	2022	68	124	Independent factors related to NEC occurrence	Prognosis of NEC treatment
Teišerskas J	2019	54	54	Independent factors related to NEC occurrence	-
Valentine GC, et al.	2019	338	238	Independent factors related to NEC occurrence	-
Yang CC, et al.	2018	29013	116052	Independent factors related to NEC occurrence	-
Zhang LP, et al.	2019	33	33	Independent factors related to NEC occurrence	-
Zeng DF	2017	59	80	Independent factors related to NEC occurrence	Prognosis of neonates with NEC after surgical intervention
Ceng SY	2021	30	467	Independent factors related to NEC occurrence	Occurrence of complications
Chen W	2021	25	25	Independent factors related to NEC occurrence	-
Cheng SP	2016	66	132	Independent factors related to NEC occurrence	-
Cui GH	2019	70	140	Independent factors related to NEC occurrence	-
Fan WT	2019	69	100	Independent factors related to NEC occurrence	Postoperative complications and death outcomes
Fan YZ	2015	63	70	Independent factors related to NEC occurrence	Incidence of NEC
Hou AN	2017	76	80	Independent factors related to NEC occurrence	Occurrence of complications
Huang YQ	2017	26	534	Independent factors related to NEC occurrence	Incidence of NEC
Jiang CC	2020	70	84	Independent factors related to NEC occurrence	-
Ke H	2017	32	96	Independent factors related to NEC occurrence	-
Li HY	2014	98	80	Independent factors related to NEC occurrence	-
Li LQ	2006	20	80	Independent factors related to NEC occurrence	Effect of probiotics on NEC prevention
Li MQ	2019	28	28	Independent factors related to NEC occurrence	-
Li XH	2019	25	175	Independent factors related to NEC occurrence	Clinical characteristics of NEC
Liu YX	2021	46	1127	Independent factors related to NEC occurrence	-
Liu YC	2019	95	2196	Independent factors related to NEC occurrence	Perinatal data of two groups of parturients
Lu XY	2013	54	57	Independent factors related to NEC occurrence	-
Lu Y	2022	52	52	Independent factors related to NEC occurrence	-
Lu M	2015	59	95	Independent factors related to NEC occurrence	-
Ma J	2019	41	383	Independent factors related to NEC occurrence	-
Ma XJ	2021	54	106	Independent factors related to NEC occurrence	Clinical symptoms of NEC children
Ma ZX	2017	36	182	Independent factors related to NEC occurrence	-
Shang Y	2014	37	62	Independent factors related to NEC occurrence	-
Shi Y	2019	32	150	Independent factors related to NEC occurrence	-
Sun HX	2017	37	60	Independent factors related to NEC occurrence	-
Tao Y	2012	63	126	Independent factors related to NEC occurrence	Occurrence of complications
Wang B	2019	50	72	Independent factors related to NEC occurrence	-
Wang J	2021	95	95	Independent factors related to NEC occurrence	Incidence of perinatal complications of mother of NEC children, occurrence of complications of NEC children, and comparison of inflammatory indicators between early NEC group and diagnosed NEC group
Wang PP	2020	30	34	Independent factors related to NEC occurrence	-
Wang WH	2013	51	120	Independent factors related to NEC occurrence	-
Wang XQ	2017	58	36	Independent factors related to NEC occurrence	-
Wang Y	2017	85	2678	Independent factors related to NEC occurrence	-
Wang ZQ	2020	42	38	Independent factors related to NEC occurrence	-
Xi E	2017	60	60	Independent factors related to NEC occurrence	Risk factors influencing mortality of NEC patients
Xu LY	2018	247	191	Independent factors related to NEC occurrence	Efficacy of probiotics on NEC
Yu M	2018	146	146	Independent factors related to NEC occurrence	Prognosis of children with NEC treated with different methods
Zhang L	2017	61	376	Independent factors related to NEC occurrence	Prognosis of NEC children (mortality, microcephaly, severe neurodevelopmental problems)
Zhao XH	2017	46	78	Independent factors related to NEC occurrence	-
Zhou XM	2017	67	73	Independent factors related to NEC occurrence	Occurrence of complications
Zhu JL	2020	38	462	Independent factors related to NEC occurrence	Occurrence of complications
Zhuang XY	2007	20	80	Independent factors related to NEC occurrence	Effectiveness of probiotics in preventing NEC in premature infants and changes in body mass
Zou YM	2021	50	45	Independent factors related to NEC occurrence	Occurrence of complications
